# Progress in Human Embryonic Stem Cell Research in the United States between 2001 and 2010

**DOI:** 10.1371/journal.pone.0120052

**Published:** 2015-03-26

**Authors:** Keyvan Vakili, Anita M. McGahan, Rahim Rezaie, Will Mitchell, Abdallah S. Daar

**Affiliations:** 1 London Business School, London, United Kingdom; 2 Rotman School of Management, University of Toronto, Toronto, Canada; 3 Munk School of Global Affairs, University of Toronto, Toronto, Canada; 4 Asia Pacific Foundation of Canada, Vancouver, Canada; 5 Sandra Rotman Centre, University of Toronto and University Health Network, Toronto, Canada; 6 Dalla Lana School of Public Health and Dept. of Surgery, University of Toronto, Toronto, Canada; 7 Stellenbosch Institute for Advanced Study, Wallenberg Research Centre, University of Stellenbosch, Stellenbosch, South Africa; Merck & Co., UNITED STATES

## Abstract

On August 9th, 2001, the federal government of the United States announced a policy restricting federal funds available for research on human embryonic stem cell (hESCs) out of concern for the “vast ethical mine fields” associated with the creation of embryos for research purposes. Until the policy was repealed on March 9th, 2009, no U.S. federal funds were available for research on hESCs extracted after August 9, 2001, and only limited federal funds were available for research on a subset of hESC lines that had previously been extracted. This paper analyzes how the 2001 U.S. federal funding restrictions influenced the quantity and geography of peer-reviewed journal publications on hESC. The primary finding is that the 2001 policy did not have a significant aggregate effect on hESC research in the U.S. After a brief lag in early 2000s, U.S. hESC research maintained pace with other areas of stem cell and genetic research. The policy had several other consequences. First, it was tied to increased hESC research funding within the U.S. at the state level, leading to concentration of related activities in a relatively small number of states. Second, it stimulated increased collaborative research between US-based scientists and those in countries with flexible policies toward hESC research (including Canada, the U.K., Israel, China, Spain, and South Korea). Third, it encouraged independent hESC research in countries without restrictions.

## Introduction

How important is public funding to science? This paper presents an analysis of the impact of restrictions implemented in the United States in 2001 on federal funding for human embryonic stem cell (hESC) research [1]. The analysis investigates how the change in funding influenced the geographic location of scientific inquiry in the burgeoning field of hESC research. Our analytical strategy compares publication trends in hESC with other areas of stem cell and genetic medicine to isolate as precisely as possible the specific impact of the U.S. federal funding change on research in hESC. The results help resolve long-standing questions [2] about whether the policy damaged U.S. global competitiveness in science, and point to the national and cross-border consequences of restrictive funding policies.

## Methods

To establish these results, we compared the locations of published hESC authors with those in two unrestricted fields: non-hESC stem cell research (i.e., ‘other SC’) and a type of genetics research called RNA interference (RNAi). Our findings are based on analysis of 79,939 articles on stem cells (SC) published between 1980 and 2010 that were reported in Scopus, an internationally recognized database of peer-reviewed scientific articles as well as 13,813 articles from 1998 to 2010 on RNAi, a parallel area of genetic science that arose at about the same time as hESC science. The identification process involved category assessment, expert review, and a comprehensive scan of all titles and abstracts across in the entire Scopus dataset. Scopus is the most comprehensive library of peer-reviewed academic publications. The peer-review process is central to the accumulation of knowledge in academic research. We report analyses based on counts of publications; the results are similar if we weight each article by the number of times it was subsequently cited (a common method for assessing article quality). The results also are robust to alternative methods for identifying SC and hESC articles using Medical Subject Headings (MeSH) categorizations. From among the SC articles, we also identified the subset of 1,847 hESC publications.

The conclusions rely on a comparison of the countries of authorship on hESC articles with those of other SC and RNAi articles. To make the comparison, we identified the country of affiliation for every author of each hESC, SC, and RNAi publication. Some publications were authored by researchers affiliated exclusively with U.S. institutions (U.S.-only) while others were authored by teams from institutions either exclusively in other countries (e.g., China) or in multiple countries (e.g., U.S. and China). For papers with authors in more than one country, the analysis credited each involved country. Separately, we categorized each country’s hESC policy as either “constrained” or “flexible” based on public records concerning policies, laws, and debates from the early 1990s through the late 2000s. Relatively constrained countries were Austria, Colombia, France, Germany, Italy, Japan, Norway, Poland, Slovakia, and Tunisia. Countries with more flexible policies were Argentina, Australia, Belgium, Brazil, Canada, Chile, China, Croatia, the Czech Republic, Denmark, Finland, Greece, Hong Kong, Hungary, Iceland, India, Iran, Israel, Mexico, the Netherlands, New Zealand, Portugal, Romania, Russia, Saudi Arabia, Singapore, South Africa, South Korea, Spain, Sweden, Switzerland, Taiwan, Turkey, and the U.K. Each country was identified as “flexible” or “constrained” category for the entire period. Judgment was required for countries that reduced constraints after initial restrictions or engaged in deep debate about guidelines. Constrained countries typically specify research on hESC to be illegal but permit research on other SC sources. The U.S. was a distinct case as generally flexible but with federal funding restrictions that constrained hESC research if alternative funding was not available.

The comparison sought to identify whether and how hESC science changed after the 2001 U.S. federal policy relative to other SC and RNAi science not targeted by similar funding restrictions. We asked: Did the global share of hESC publications by U.S.-based scholars decline relative to their share of SC and RNAi publications? How did publication levels compare across countries with flexible versus constrained policies? Did U.S. states that provided funding for hESC science account for a disproportionate increase in share of hESC publications relative to other SC and RNAi fields? Finally, did U.S.-based scholars turn to cross-border collaboration with scholars in flexible-policy countries in response to the 2001 U.S. federal funding restrictions? Generally, we sought to determine whether the US fell behind other countries in hESC research after the 2001 policy was implemented, as was speculated in the early 2000s.

## Results

**Fig 1:**
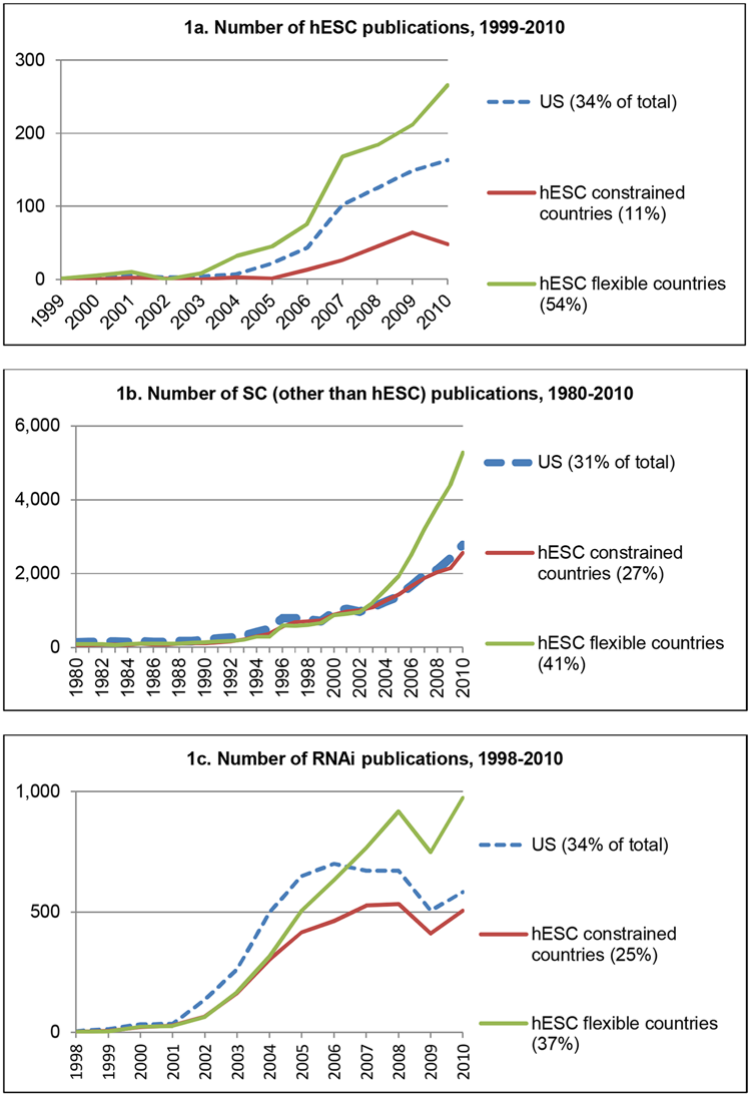
Number of Publications of hESC, Other SC, and RNAi Research by Country of Authorship. The lines in the figures report the number of publications by scholars based in the U.S. and in countries with either constrained or flexible policies regarding hESC research; publications with authors from more than one country are credited to each country. The vertical axis reports the number of publications. The percentages in parentheses denote the share of total publications during the period (the shares sum to slightly less than 100% because they do not include publications from countries with no specified hESC policies: for hESC, this amounts to the exclusion of 15 publications from scholars in seven countries). The difference in the share of hESC publications from flexible versus constrained countries is significantly greater than the differences for other SC and RNAi (p<0.05).

Fig. 1 and Table 1 show the location of hESC researchers compared to that of scientists studying other SC and RNAi. After the publication in *Science* of the first major study on hESC on November 6, 1998 [3], only a few hESC papers were published over the next five years (42 in total). After 2003, the number of published hESC studies increased dramatically first in countries with flexible policies and then in the U.S.

**Table 1:** Top Countries for hESC Research, with Comparison to Other SC and RNAi.

	hESC policies	hESC1999-2010	Other SC1980-2010	Other SC1999-2010	RNAi1998-2010
**All publications**		**1,847**	**78,092**	**64,047**	**13,813**
**United States**	**Federal limits**	**34%**	**31%**	**29%**	**34%**
United Kingdom	Flexible	8%	6%	6%	6%
China	Flexible	6%	9%	11%	13%
South Korea	Flexible	5%	2%	3%	2%
Israel	Flexible	4%	1%	1%	1%
Singapore	Flexible	4%	1%	1%	1%
Germany	Constrained	4%	8%	8%	7%
Sweden	Flexible	4%	2%	2%	1%
Japan	Constrained	4%	9%	9%	10%
Canada	Flexible	4%	3%	3%	3%
Australia	Flexible	3%	2%	2%	1%
Spain	Flexible	3%	2%	2%	1%
France	Constrained	2%	4%	4%	4%
Netherlands	Flexible	2%	3%	2%	2%
India	Flexible	2%	1%	1%	1%
Italy	Constrained	1%	4%	4%	2%
	**All constrained**	**11%**	**27%**	**28%**	**25%**
	**All flexible**	**54%**	**41%**	**43%**	**37%**

The percentages report national shares of publications for hESC, other SC, and RNAi research, based on countries in which authors are based. For “Other SC”, we report both 1980–2010 (the full period of SC publications) and 1999–2010 (concurrent with the start of hESC publication). The results demonstrate that hESC scientists in constrained countries lost ground to those in flexible countries, while U.S.-based scientists maintained at least as strong a position in hESC as in the other fields.

Fig. 1a shows that hESC publications by U.S.-based scientists grew steadily during the 2000s, although with an initial lag behind more flexible countries until state-level funding became available in 2005. Publications in the 34 countries with flexible hESC policies grew early and quickly while publications from the 10 hESC-constrained countries lagged. Despite the greater aggregate publications from flexible countries, the U.S. maintained a substantial lead. Table 1 lists the top sixteen countries for hESC publications (87% of all publications); the U.K. took second with 8%, well behind the 34% U.S. share.

Would U.S. hESC scholars have been more active if federal funding restrictions had not been enacted? To address this question, we examine the U.S. share of other SC and RNAi research. Any relatively larger U.S. share of other SC and/or RNAi research would suggest that the 2001 federal policy restricted hESC research. Fig. 1b and 1c reject this hypothesis. Despite an early dampening, U.S. scientists published hESC studies at similar rates to other SC and RNAi studies over the 2000s. Furthermore, the global share of hESC publications by U.S.-based researchers (34%) was similar to both comparison groups (other SC at 31% and RNAi at 34%). Moon and Cho (2014) [4] have recently reported that the 2001 U.S. federal policy did in fact diminish hESC research on aspects that relate to *derivation* of stem cell lines, as compared to other types of hESC research that focused on differentiation and medical applications. However, three regularities indicate that any potentially adverse impact of the 2001 federal policy was likely mitigated so as not to have a clearly discernable impact on overall hESC publication trends: first, the low extent of the reduction (6.12%) in derivation research by U.S.-based scientists relative to their overall hESC performance; second, that derivation research constitutes a minority (<10%) of hESC publications; and third, that derivation research was a particularly active area for international collaboration by U.S.-based scientists.

The importance of institutions governing hESC research is evident in analysis of other countries in which hESC science was constrained by explicit policies or by prevailing cultural norms (i.e., ‘constrained countries’ such as Germany, France and Japan). Fig. 1a shows that hESC research in constrained countries strongly trailed the U.S., whereas Fig. 1b and 1c demonstrate that constrained countries maintained shares in other SC and RNAi science. By contrast, flexible countries gained more share than constrained countries in hESC than in other SC and RNAi. Hence, constrained countries lost publication share in hESC relative to both the U.S. and flexible countries. Germany and Japan are the only constrained countries in the top 10 of hESC science, but with hESC shares that are substantially lower than their shares of other SC and RNAi research.

It is important to note that, while federal hESC funding restrictions in the U.S. did not deflate overall scholarly activity by U.S.-based researchers, important changes occurred nonetheless. First, as Moon and Cho (2014) reported, the 2001 U.S. federal policy did in fact diminish U.S. research performance in the type of research that it targeted; namely *derivation* research that would lead to new embryonic cell lines. Second, as we show below, the effects of the 2001 policy may have been mitigated by increases in funding in some U.S. states and by greater cross-border collaborations between US and non-US researchers.

**Fig 2:**
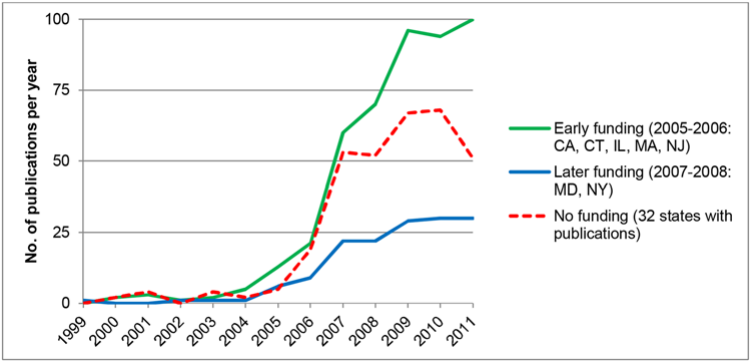
hESC Publication Trends in U.S. States With and Without Funding, 1999–2011. The vertical axis in the figure reports the number of publications per year in three classes of states: States in which funding for hESC research began in 2005–2006 (green line), those in which funding began in 2007–2008 (blue line), and those that never funded hESC research (red line).

To investigate these possibilities, we compared publications in U.S. states with and without support for hESC research. Total state-level support was extensive. In 2007, for example, U.S. states provided $250 million for hESC research [5]. Fig. 2 reports on five “early funding” states (2005–2006: CA, CT, IL, MA, NJ), two “later funding” states (2007–2008: MD, NY), and the remaining “no funding” states. In addition, there are indications that federal restrictions were associated with increases in private-sector funds for research in some states [6]. Publication trends across the three groups (i.e., early, late, and no funding states) were similar until about 2007, when they diverged sharply. Publication counts in early funding states escalated while counts in no funding states declined. Researchers in later funding states initially lost ground but recovered after funding was implemented. The net effect was a shift toward the concentration of U.S. hESC science in a small number of U.S. states. California (30% of publications) and Massachusetts (12%) were particularly important. While the funding, in part, likely reflected the historical research in each state, the divergent pattern in publication growth trajectories after 2005 is consistent with the change in funding by state. This change suggests that state funds effectively substituted, at least to some degree, for funding gaps at the federal level.

**Fig 3:**
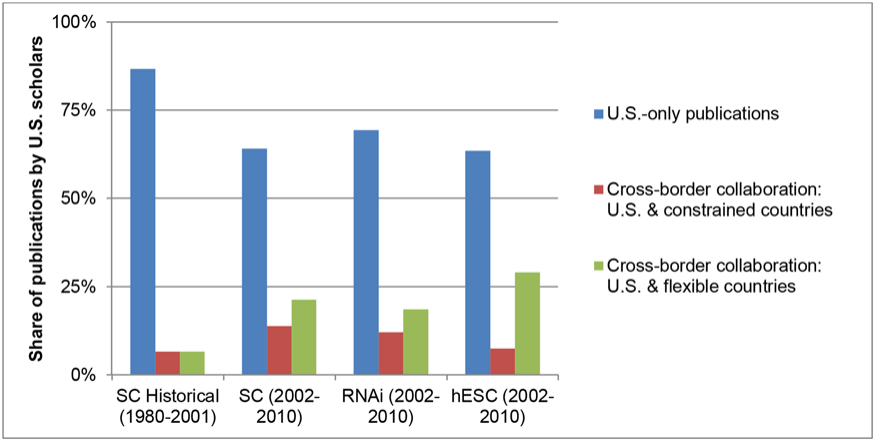
U.S.-only Publications and U.S.-Cross-Border Collaborations. The figure reports the share of publications by U.S.-based scientists that involved only U.S.-based researchers and those with co-authors based in other countries. The categories include historical patterns for SC before the U.S. federal funding limits (1980–2001), as well as for SC (other than hESC), RNAi, and hESC after the funding limits (2002–2010).

hESC research outside the U.S. accelerated during the 2000s, particularly in flexible-policy countries (see Fig. 1a). This trend led us to investigate whether U.S. scientists responded to federal funding cuts by conducting research in partnership with scholars in flexible-policy nations. Fig. 3 shows the level of publications by authoring teams located either only in the U.S. or in both the US and elsewhere. While it shows a share decrease in U.S.-only research in both SC and hESC after 2001, perhaps reflective of the global diffusion of research skills, it also demonstrates that, after 2001, a particularly pronounced increase in cross-border collaboration between U.S. researchers and scholars in flexible-policy countries. Almost 30% of hESC publications by U.S. scientists were collaboratively authored with non-U.S. researchers. U.S. cross-border collaboration with hESC scholars in flexible countries was higher than for other SC or RNAi, while collaboration with hESC scientists in constrained countries was substantially lower than in the other two fields during the 2000s. During the 2002–2010 period, hESC scientists in the U.S. partnered with flexible-country co-authors at 3.9 times the rate as with constrained-country partners, versus only 1.5 times for other SC and RNAi. The top five preferred countries for partnering U.S.-based hESC scholars were Canada (9%), the U.K. (8%), Germany (8%), Israel (8%), and China (7%). Germany, the only constrained country in the group, had substantially less share in hESC than in other SC (12%) and RNAi (10%). Japan fell from the top two in other SC and RNAi to rank ninth among U.S. collaborators in hESC. Thus, although cross-border research was important in all areas, U.S. cross-border hESC collaboration disproportionately favored scientists in flexible-policy countries. This pattern suggests that international collaboration may have offered a hedge against domestic restrictions for U.S. hESC scientists.

One mechanism accounting for these patterns may have been signalling. The 2001 U.S. federal funding policy may have created a perception in some countries that hESC research might present an ideal area for greater investment. Dominique McMahon (2011, p. 160), focusing on regenerative medicine (RM) innovation in China, India and Brazil [7], found that: "several interviewees in each country indicated that RM was a strategic field to become involved in due to the reluctance of some countries, particularly the United States, to pursue hESC research. Interviewees from all three countries felt that these political struggles gave them a real chance to make a difference in the field." These findings are consistent with those reported by Moon and Cho (2014), who indicated that, while the U.S. share of derivation hESC publications declined significantly after 2001, researchers in flexible-policy countries enhanced their efforts in the derivation area of hESC research (achieving 5.2% greater international share in derivation research than in other areas of hESC). The latter effect, the authors reported, was especially pronounced between 2002–2005 when the share of derivation research in flexible countries was 17.8% higher than overall in hESC research. They also found that U.S. collaboration with researchers in the flexible countries disproportionally favoured derivation research. Therefore, while not oriented toward the global scientific community, the 2001 U.S. federal policy does appear to have had international ramifications. Moon and Cho (2014) conclude that: “the U.S. scientific community showed prominent resilience in hESC research through international collaboration.” We extend this notion to suggest that institutional responses at both the state level and internationally amount to a collective *scientific resilience* that mitigates the impact of national policies.

## Discussion

Our results suggest a complex interplay between the geography of science, moral considerations, and public policy. The analysis demonstrates that, contrary to concerns expressed at the time, the 2001 U.S. federal funding constraints did not have a significant impact on aggregate levels of hESC research within the country. We have argued that this result may have more to do with mitigating factors than the policy’s lack of potential for impact. The policy’s main effect was to shift the geography of hESC research into states and countries with regimes and funding more favourable to hESC research. The results point to the resilience of the scientific enterprise at both local and international levels. Attempting to shape scientific inquiry in a specific geographic area may drive the targeted activity into locations with fewer restrictions and/or more support. In this respect, the 2001 U.S. hESC policy had considerable consequences in publishable research, some of which may have bene unforeseen and unintended. These consequences may also have carried implications for downstream phenomenon such as patenting, drug development, and licensing, for which additional research is warranted.
